# Self‐reporting and screening: Data with right‐censored, left‐censored, and complete observations

**DOI:** 10.1002/sim.9434

**Published:** 2022-05-24

**Authors:** Jonathan Yefenof, Yair Goldberg, Jennifer Wiler, Avishai Mandelbaum, Ya'acov Ritov

**Affiliations:** ^1^ Statistics and Data Science The Hebrew University of Jerusalem Jerusalem Israel; ^2^ The Faculty of Industrial Engineering and Management Technion ‐ Israel Institute of Technology Haifa Israel; ^3^ School of Medicine University of Colorado Boulder Colorado USA; ^4^ Department of Statistics University of Michigan Ann Arbor Michigan USA; ^5^ Present address: Department of Statistics The Hebrew University of Jerusalem Jerusalem Israel.

**Keywords:** current status data, left censoring, nonparametric estimation, right censoring, survival analysis

## Abstract

We consider survival data that combine three types of observations: uncensored, right‐censored, and left‐censored. Such data arises from screening a medical condition, in situations where self‐detection arises naturally. Our goal is to estimate the failure‐time distribution, based on these three observation types. We propose a novel methodology for distribution estimation using both semiparametric and nonparametric techniques. We then evaluate the performance of these estimators via simulated data. Finally, as a case study, we estimate the patience of patients who arrive at an emergency department and wait for treatment. Three categories of patients are observed: those who leave the system and announce it, and thus their patience time is observed; those who get service and thus their patience time is right‐censored by the waiting time; and those who leave the system without announcing it. For this third category, the patients' absence is revealed only when they are called to service, which is after they have already left; formally, their patience time is left‐censored. Other applications of our proposed methodology are discussed.

## 1 INTRODUCTION

We study the estimation of failure time distribution where the failure times can be either observed directly, or be right‐censored or left‐censored. This type of survival data arises, for example, in estimation of time to the appearance of a medical condition where characteristic symptoms may or may not appear when the condition exists. Specific medical settings include relapse in childhood brain tumors, which may be observed due to clinical symptoms, or right‐censored due to periodic screening with negative result (no tumor), or left‐censored due to periodic screening with a positive result.^1^ Another medical setting is melanoma cancer, which is observed if self‐detected, or is right censored due to a negative screening (no melanoma), or left‐censored if it goes undetected until screening. Additional examples can be found in Whitehead.^2^

The motivating example for this work comes from estimating customer patience in service system which is a challenging problem.^3^ In our study, we focus on patients who wait for treatment in an emergency department (ED). Three categories of patients are observed. The first category consists of patients who get service and thus their patience time is right‐censored by the waiting time. The second category comprises those who leave the system and announce it, and thus their patience time is observed while the waiting time is right‐censored. The third category consists of patients who leave the system without announcing it; their absence is hence revealed only when they are called to service, which is after they have already left; formally, their patience time is left‐censored. Note that the data structure is a special case of interval‐censored data.^4^ Here, interval‐censored data is a general data structure which many popular survival data settings are special cases of, including both right‐censored data and left‐censored data.^5^ The specific setting that is considered here includes both left‐censored and right‐censored observations as well as complete observations.

Estimating the patience time is of importance as the decision of patients to leave the system before getting served might have a strong effect on their physical well‐being. There has been considerable research on the reasons why patients leave an ED before being served.^6^, ^7^, ^8^, ^9^ However, these and other authors have not proposed a model by which ED patience time—namely the duration that a potential patient is willing to wait for ED service—can be estimated, and this is our goal here.

We propose novel semiparametric and nonparametric estimators of the unknown survival function for this 3‐type survival data. We then study their rates of convergence. The semiparametric estimator is based on both full and partial likelihoods. We provide condition under which the semiparametric estimator is a linear asymptotic normal (LAN) estimator and converges to a normal distribution in a root‐n rate. The nonparametric estimator is based on nonparametric kernel estimators for density functions and on a novel estimator of the cumulative probability function that has some similarities to the Nelson‐Aalen estimator.^10^ We show that, under some regularity conditions, the nonparametric estimator point‐wise converges to the normal distribution.

We perform a simulation study and compare the proposed semiparametric and nonparametric estimators. For the semiparametric model, we study both correct and misspecified models and show the different corresponding results. We show how the accuracy changes with sample size. We then carry out a case study that is based on data of patients waiting for treatment in an ED, in the U.S. in 2008. We analyzed separately different severity levels (15 106 observations in the emergency group, 43 600 in the urgent group, and 26 541 in the semi‐urgent group). We conclude with a comparison of the semiparametric and nonparametric estimators for the three different severity levels of this dataset.

## 2 BRIEF LITERATURE REVIEW

Developing screening methods for medical conditions, such as breast and melanoma cancers, has a long history.^11^, ^12^ In the classical setting, the medical condition either already exists at the time of screening and is thus left‐censored, or does not exist, and is thus right‐censored. The setting in which self‐detection is possible, and thus the condition time is observed, has been surprisingly mostly ignored in the literature. For example, Minn et al^1^ treat both self‐detection times and screening times as event times, ignoring the censoring. The closest model to the one that we present here appears in Whitehead.^2^ It is assumed there that the condition can be detected at screening or before screening due to symptoms. In both cases, the condition already exists at the time of detection. It is also assumed that screenings take place at a sequence of fixed time points. Whitehead^2^ recommends to ignore the extra knowledge gained due to self‐reporting and to replace these times with the time of the next screening. The survival function is then estimated only at the discrete fixed screening times using standard techniques.^13^

There has been considerable research effort, dedicated to modeling and analysis of customer (im)patience while waiting for service. Here we describe several papers that, together with references therein, provide what is required for a historical background and state‐of‐art perspective. First, we recommend the recent literature review^9^ (Section 3) in Batt and Terwiesch, accompanied by Gans et al.^14^ These survey patience‐research from an operational/queuing view point (mainly section 6.3.3 in the latter), while connecting it to the medical literature on patients who are left without being seen (LWBS) (mainly Section 3 in the former); see also Aksin et al^15^ who expand on managerial challenges. Next we mention Mandelbaum and Zeltyn,^16^ which is an Explanatory Data Analysis of (im)patience in telephone call centers (that appears in a special issue that is devoted to models of queues abandonment). Finally, and the most related to the present study, are the following two studies. Brown et al^17^ applies, in Section 5, the Kaplan‐Meier estimator^18^ to estimate the survival functions and consequently hazard rates, of both virtual waiting time and impatience; the data is that of a call center, in which times of abandonment are all recorded hence the data is right‐censored. Then Wiler et al,^19^ which is also the source of our present ED data case study, estimate LWBS rates as a function of ED patient arrival rates, treatment times, and ED boarding times. There was no attempt in that work to estimate the patience‐time distribution.

We conclude this brief survey with the observation that the estimation of customer (im)patience is relevant beyond screening, call centers, and EDs. For example, Nah^20^ studies tolerance of Web users (during information retrieval). Yom‐Tov et al^21^ analyzes chat services, in which customers abandon at any phase during chat‐exchanges with a service center: one expects that such services give rise to the same options as in EDs: some customers receive service, others abandon without letting anyone know, and the rest announce their abandonment time.

## 3 THE MODEL

In the standard setting of right‐censored data one observes, for each patient, either the failure time or the censoring time. In terms of our motivating example, failure time is patience time while censoring time is the waiting time. Patience time is observed when patients leave the ED while informing the system of their departure; waiting time is observed when a patient is called for service. However, unlike in standard right‐censored data and like in current status data, there are also patients who leave without informing; in this case their absence is observed only when they are called for service, and this latter time provides an upper bound for their patience time. In other words, the (virtual) waiting time is observed, and the only information on patience time is that it is less than this observed waiting time. Hence, in this case, the patience time is left‐censored.

More formally, let T be the patient's failure time, that is, the time until the patient loses patience. Let W be the censoring time, that is, the waiting time until the patient gets (or could have gotten) service. We assume that T has a cumulative distribution function (cdf) F and a probability density function (pdf) f, and that W has cdf G and pdf g. Let Δ be the indicator Δ≡1{T<W}; that is, Δ=1 if the patient loses patience before being called to service, and Δ=0 otherwise.

Let Y be the indicator that is 1 for a patient who leaves and informs when leaving, and 0 otherwise. Denote by q(t) the conditional probability that a patient reports leaving given that the waiting time equals to t. In other words, q(t)=pr(Y=1|T=t). The assumption that patience time T and waiting time W are independent is common in survival analysis, for example, when using the Kaplan‐Meier estimator.^18^ Since T and W may be dependent, one can use strata to overcome this challenge as was done in the case study in Section 7. The announcement indicator Y depends on the time through the function q(t). In other words, given the patience time T=t, the decision on announcement does not depend on actual waiting time W. However, due to censoring, the decision on the announcement is observed only when t<W. Summarizing, we assume that the pair (Y,T) is independent of the waiting time W. When this assumption does not hold, different theoretical tools are needed for a valid estimation.

Let U be the recorded time: U≡YT+(1−Y)W. The observed data consist of the triplets $\left(U_{i},Y_{i},\Delta_{i}\right)$, i=1,…,n, and there are three categories of patients:

𝒞=1: The patient gets service, hence the waiting time is observed, which serves as a lower bound on the patience time; thus the patience time is right censored. Formally, Δ=0, Y=0, and U=W.
𝒞=2: The patient leaves without being treated and reports departure. The patience time is thus revealed: Y=1, Δ=1, and U=T.
𝒞=3: The patient leaves without reporting, hence virtual waiting time (the time that the patient would have waited had he stayed in the ED) is observed, which provides an upper bound for the patience time, thus the patience time is left‐censored. Formally, Y=0, Δ=1, and U=W.

**FIGURE 1:**
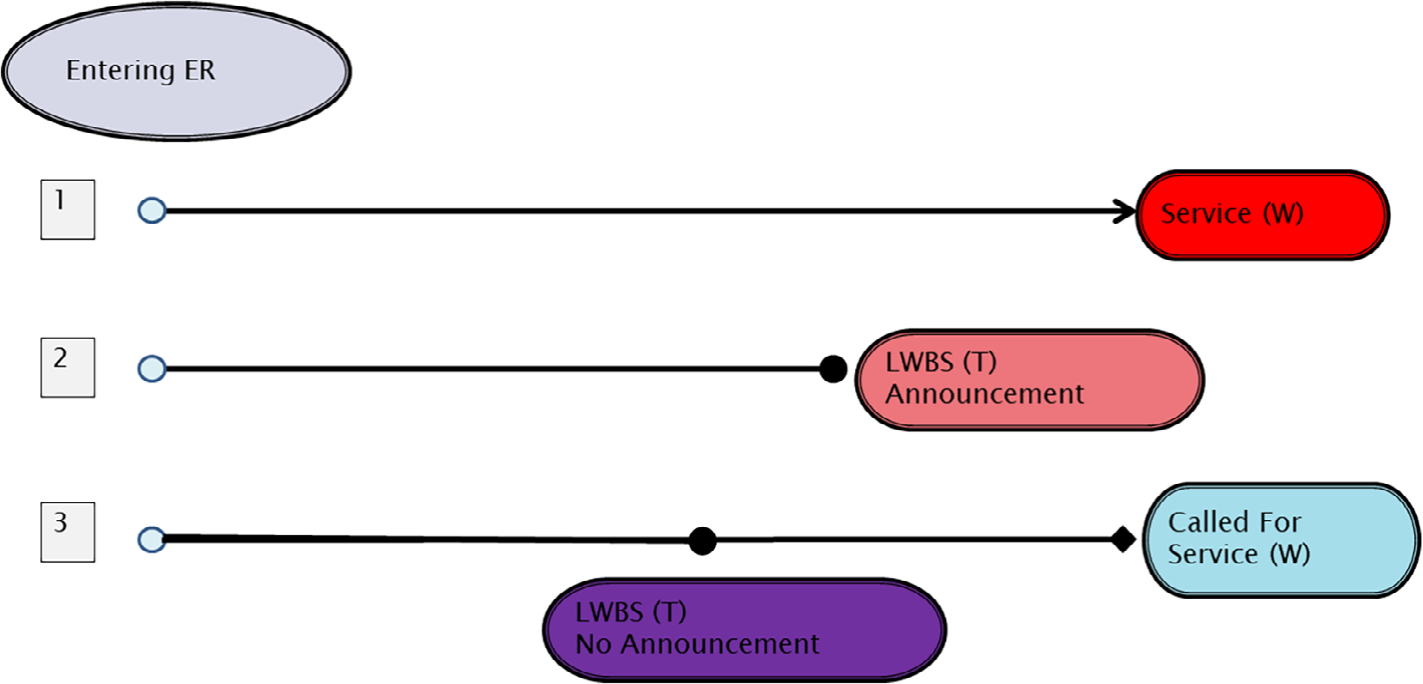
The three patient categories. Category 1 includes patients that received service. Category 2 includes patients that left without being seen and announced before leaving. Category 3 includes patients that left without being seen but did not announce leaving

A graphical diagram of these categories appears in Figure 1.

Lemma 1 *The following equalities hold*:

i)
$pr(U\leq t,𝒞=1)=\int_{0}^{t}g(w)\bar{F}(w)dw$.
ii)
$pr(U\leq t,𝒞=2)=\int_{0}^{t}q(w)f(w)\bar{G}(w)dw$.
iii)
$pr(U\leq t,𝒞=3)=\int_{0}^{t}g(w)\int_{0}^{w}\left\{1-q(x)\right\}f(x)dxdw$.

Here, $\bar{F}(t)=1-F(t)$ and $\bar{G}(t)=1-G(t)$ are the survival functions of the patience time and the waiting time, respectively.

See the proof in Appendix A.1.

For i=1,2,3, we introduce the following sub‐stochastic density functions

(1) $$h_{i}(t):=\frac{d}{dt}pr(U\leq t,𝒞=i).$$

From Lemma 1 above, we deduce that

$$h_{1}(t)=g(t)\bar{F}(t),\;\;h_{2}(t)=q(t)f(t)\bar{G}(t),\;\;h_{3}(t)=g(t)\int_{0}^{t}\left\{1-q(x)\right\}f(x)dx.$$

Define

(2) $$r_{1}(t)\equiv \frac{h_{1}(t)}{pr(W\leq T)},\;r_{2}(t)\equiv \frac{h_{2}(t)}{pr(Y=1,W>T)},\;r_{3}(t)\equiv \frac{h_{3}(t)}{pr(Y=0,W>T)}.$$

Then $r_{i}$ is the density function of the observed time U given 𝒞=i. Our model assumes that all denominators are positive.

To summarize, what is known and what is to be estimated, there are two unknown distributions in our setting, G and F, and we aim to estimate them using both semiparametric and nonparametric techniques. For each patient, the waiting time is either observed or right censored. If the patient reports and then leaves, the waiting time is longer than the observed patience time. Hence, the waiting time is right‐censored. Therefore, semiparametric and nonparametric estimation for the distribution of waiting time W can be done by standard techniques for right‐censored data. However, estimation of the distribution of patience time T, is more complicated and is discussed in Sections 4 and 5.

## 4 SEMIPARAMETRIC ESTIMATION

Assume now that the distributions of both the patience time and the waiting time belong to some parametric families. More formally, let ℱ={f(·;θ),θ∈Θ} where $\Theta \subseteq {\mathbb{R}}^{d}$, 𝒢={g(·;γ),γ∈Γ} where $\Gamma \subseteq {\mathbb{R}}^{p}$. We assume that the density of the patience time can be written as $f(t;\theta_{0})\in ℱ$. We also assume that the density of the waiting time can be written as $g(t;\gamma_{0})\in 𝒢$. Write $h_{1}(t;\theta ,\gamma )\equiv g(t;\gamma )\bar{F}(t;\theta )$, and similarly $h_{2}(t;\theta ,\gamma )\equiv q(t)f(t;\theta )\bar{G}(t;\gamma )$ and $h_{3}(t;\theta ,\gamma )\equiv g(t;\gamma )\int_{0}^{t}\left\{1-q(x)\right\}f(x;\theta )dx$.

The likelihood of the observed data $D=\{(U_{i},Y_{i},\Delta_{i}),i=1,\ldots ,n\}$ can be written in terms of the functions $h_{1}$, $h_{2}$, and $h_{3}$, as follows:

$$L(D;\theta ,\gamma )=\overset{n}{\underset{i=1}{\prod }}{\left\{h_{1}(U_{i};\theta ,\gamma )\right\}}^{1-\Delta_{i}}\left\{h_{2}(U_{i};\theta ,\gamma )\right\}{}^{\Delta_{i}Y_{i}}\left\{h_{3}(U_{i};\theta ,\gamma )\right\}{}^{\Delta_{i}(1-Y_{i})}.$$

Using the explicit representations of $h_{1}$, $h_{2}$, $h_{3}$, we obtain that L(D;θ,γ) is given by

$$\begin{array}{ll} \overset{n}{\underset{i=1}{\prod }} & \left(\left\{g(U_{i};\gamma )\bar{F}(U_{i};\theta )\right\}{}^{1-\Delta_{i}}\left\{q(U_{i})f(U_{i};\theta )\bar{G}(U_{i};\gamma )\right\}{}^{\Delta_{i}Y_{i}}\right.\\ & \times \left.{\left[g(U_{i};\gamma )\int_{0}^{U_{i}}\left\{1-q(s)\right\}f(s;\theta )ds\right]}^{\Delta_{i}(1-Y_{i})}\right). \end{array}$$

The value of γ that maximizes this likelihood is independent of θ. Therefore, a maximum likelihood estimator (MLE) ${\hat{\gamma }}_{n}$ to $\gamma_{0}$ can be constructed from this likelihood.

Maximizing the likelihood with respect to θ is difficult. Even if $\gamma_{0}$ is given or estimated, the maximizer of θ depends on the unknown function q(t). To address this challenge, we propose using a partial likelihood approach^22^ which avoids the need to estimate q(t). The partial likelihood that we use here is the likelihood calculated only for a specific category while ignoring the data for the other categories. In Theorem 1 below we show that, under standard regularity conditions, the maximizer of the partial likelihood is a consistent and asymptotically normal estimator for $\theta_{0}$.

We consider the partial likelihood $L_{partial}(D;\theta ;\gamma )$ of category 𝒞=1,

$$\overset{n}{\underset{i=1}{\prod }}{\left\{\frac{g(U_{i};\gamma )\bar{F}(U_{i};\theta )}{\int_{0}^{\infty }g(s;\gamma )\bar{F}(s;\theta )ds}\right\}}^{1-\Delta_{i}}.$$

The value of θ that maximizes this partial likelihood depends on γ. We plug the MLE ${\hat{\gamma }}_{n}$ into this partial likelihood. Clearly, the resulting estimator for θ does not depend on the function q(t) and thus no estimation of q(t) is needed. Finding an estimator for the announcement probability function q(t) is an interesting and challenging research question that is beyond the scope of this article.

We need the following assumptions:

(A1) The derivative $\frac{\partial }{\partial \theta }f(t;\theta )$ is continuous in t for each θ∈Θ, $\frac{\partial }{\partial \gamma }g(t;\gamma )$ is continuous in t for each γ∈Γ.
(A2) For all θ∈Θ, $\arg\max_{\gamma \in \Gamma }L(D;\theta ,\gamma )$ is unique, hence denote
$\hat{\gamma }(\theta )\equiv \arg\max_{\gamma \in \Gamma }L(D;\theta ,\gamma )$. It is assumed as well that for each θ∈Θ, $\frac{\partial }{\partial \gamma }L\left\{D;\theta ,\hat{\gamma }(\theta )\right\}=0$.
(A3) For all γ∈Γ, $\arg\max_{\theta \in \Theta }L_{partial}(D;\theta ,\gamma )$ is unique, hence denote
$\hat{\theta }(\gamma )\equiv \arg\max_{\theta \in \Theta }L_{partial}(D;\theta ,\gamma )$. It is assumed as well that for each γ∈Γ, $\frac{\partial }{\partial \theta }L_{partial}\left\{D;\hat{\theta }(\gamma ),\gamma \right\}=0$.

Theorem 1 *Let* ${\hat{\gamma }}_{n}$ *be the maximizer of* L(D;θ;γ) *and let* ${\hat{\theta }}_{n}$ *be the maximizer of* $L_{partial}(D;\theta ;{\hat{\gamma }}_{n})$ *. Then, as* n→∞ *,*

i)
${\hat{\gamma }}_{n}\to \gamma_{0}$ *in probability*.
ii)
$\sqrt{n}\left({\hat{\gamma }}_{n}-\gamma_{0}\right)\to N\left(0,V_{\gamma_{0}}\right)$ *in distribution*.
iii)
${\hat{\theta }}_{n}\to \theta_{0}$ *in probability*.
iv)
$\sqrt{n}\left({\hat{\theta }}_{n}-\theta_{0}\right)\to N\left(0,S_{\theta_{0},\gamma_{0}}\right)$ *in distribution*.

*Here* $V_{\gamma_{0}}$ *,* $S_{\theta_{0}},\gamma_{0}$ *are covariance matrices as defined in Appendix* *A.1*.

The proof appears in Appendix A.1.

Example 1 Assume that T follows an exponential distribution with rate θ and W follows an exponential distribution with rate γ. Then

$$\begin{array}{ll} {\hat{\gamma }}_{n} & =\frac{n-\sum_{i=1}^{n}\Delta_{i}Y_{i}}{\sum_{i=1}^{n}U_{i}}\\ {\hat{\theta }}_{n} & =\frac{\sum_{i=1}^{n}(1-\Delta_{i})}{\sum_{i=1}^{n}U_{i}(1-\Delta_{i})}-{\hat{\gamma }}_{n}=\frac{\sum_{i=1}^{n}(1-\Delta_{i})}{\sum_{i=1}^{n}U_{i}(1-\Delta_{i})}-\frac{n-\sum_{i=1}^{n}\Delta_{i}Y_{i}}{\sum_{i=1}^{n}U_{i}}. \end{array}$$

The details of the computation appears in Appendix A.5.

## 5 NONPARAMETRIC ESTIMATION

In this section we propose nonparametric estimators for the survival function of the patience time $\bar{F}$ and study its theoretical properties. For simplicity, we restrict the estimation to an interval [0,τ] for some τ>0, such that the probability of W and T being larger than τ is positive. This is a standard condition in survival estimation^23^ (chapter 4.2). Note that for observations of Categories 1 and 3, the waiting‐time is observed. For Category 2, only a lower bound of the waiting time is observed. Hence, the waiting time is either observed or right‐censored. Therefore, estimating the waiting time distribution can be done by using standard survival analysis estimators such as the Kaplan‐Meyer estimator. On the other hand, estimating the distribution of the patience time is more challenging since we cannot distinguish between the density function f and the unknown function q. Our goal is thus to estimate the distribution of the patience time F.

Assume that over all positive numbers, the waiting time density function g is strictly positive. Recall that $h_{1}(t)=g(t)\bar{F}(t)$, $h_{3}(t)=g(t)\int_{0}^{t}\left\{1-q(s)\right\}f(s)ds$, where the functions $h_{1},h_{3}$ are defined as in (1). Therefore,

(3) $$\frac{h_{1}(t)}{h_{3}(t)}=\frac{\bar{F}(t)}{F(t)-\int_{0}^{t}q(s)f(s)ds}.$$

which is well defined as g(t)>0. Reordering the terms in (3), we get that

$$\left\{F(t)-\int_{0}^{t}q(s)f(s)ds\right\}\frac{h_{1}(t)}{h_{3}(t)}=1-F(t).$$

Hence,

$$F(t)=\frac{h_{3}(t)+h_{1}(t)\int_{0}^{t}q(s)f(s)ds}{h_{3}(t)+h_{1}(t)}.$$

From the definitions in (2), it follows that

(4) $$F(t)=\frac{pr(Y=0,T<W)r_{3}(t)+pr(W\leq T)r_{1}(t)\int_{0}^{t}q(s)f(s)ds}{pr(Y=0,T<W)r_{3}(t)+pr(W\leq T)r_{1}(t)}.$$

Therefore, we propose to estimate F(t) by estimating the following terms:

(i) pr(W≤T) and pr(Y=0,T<W),
(ii) $r_{1}(t)$ and $r_{3}(t)$,
(iii) $A(t)\equiv \int_{0}^{t}q(s)f(s)ds$.

Estimating the expression in (i) can be done by the empirical estimators: $\hat{pr}(T\leq W)=n^{-1}\sum_{i=1}^{n}(1-\Delta_{i})$, $\hat{pr}(Y=0,W<T)=n^{-1}\sum_{i=1}^{n}\Delta_{i}(1-Y_{i})$. These estimators converge, by the central limit theorem (CLT), to pr(W≤T) and pr(Y=0,T<W), respectively, at the rate of $n^{1/2}$.

Since $r_{1}$ and $r_{3}$ are density functions, they can be estimated using a kernel estimator^24^ (chapter 1.2). Let ${\hat{r}}_{1}$ and ${\hat{r}}_{3}$ be kernel estimators of $r_{1}$ and $r_{3}$, respectively. Assume that both $r_{1}$ and $r_{3}$ belong to a Sobolev function class of order β. Then for each t>0, both ${\hat{r}}_{1}(t)$ and ${\hat{r}}_{3}(t)$ converge at a rate of $n^{\beta /(2\beta +1)}$. Here, the parameter β≥1 is an integer that represents the smoothness of a function. Specifically, if β>k for some integer k, then the function is at least k‐time differentiable.^24^

We now turn to estimate the term $A(t)=\int_{0}^{t}q(s)f(s)ds$. A nonparametric estimator that we created for this term is defined and proven to be consistent in the following lemma.

Lemma 2 *Let*

$${\hat{N}}_{n}(t)\equiv \frac{1}{n}\overset{n}{\underset{i=1}{\sum }}Y_{i}\Delta_{i}1\{U_{i}\leq t\},\;{\hat{Y}}_{n}(t)\equiv \frac{1}{n}\overset{n}{\underset{i=1}{\sum }}1\{U_{i}\geq t\}.$$

*Define* ${\hat{D}}_{n}(t)\equiv \int_{0}^{t}\frac{d{\hat{N}}_{n}(s)}{{\hat{Y}}_{n}(s)}$ *. Then* $\hat{A}(t)\equiv 1-\exp\left\{-{\hat{D}}_{n}(t)\right\}$ *converges pointwise to* A(t) *, at a rate of* $n^{1/2}$ *, for every* t∈[0,τ].

The proof is given in Appendix A.3.

By plugging in the estimators

$$\hat{pr}(Y=0,W<T),\;\hat{pr}(T\leq W),\;{\hat{r}}_{3}(t),\;{\hat{r}}_{1}(t),\;\hat{A}(t),$$

to the equation in (4), we get that

(5) $${\hat{F}}_{n}(t)=\frac{\hat{pr}(Y=0,W<T){\hat{r}}_{3}(t)+\hat{pr}(T\leq W){\hat{r}}_{1}(t)\hat{A}(t)}{\hat{pr}(Y=0,W<T){\hat{r}}_{3}(t)+\hat{pr}(T\leq W){\hat{r}}_{1}(t)},$$

is an estimator of F(t).

Theorem 2 *The estimator* ${\hat{F}}_{n}(t)$ *converges pointwise to* F(t) *at a rate of* $n^{\beta /(2\beta +1)}$ *, for every* t∈[0,τ].

The proof appears in Appendix A.4. Since that ${\hat{F}}_{n}$ is based on density estimation, it is not necessarily monotonic, we therefore replace it with a monotonic approximation. The monotonic approximation is by taking the cumulative sup.

## 6 SIMULATIONS

We study the performance of both the semiparametric and nonparametric estimators that were proposed in Sections 4 and 5, respectively. Based on the setting of the case study discussed in Section 7, we consider two simulation settings. In the case study, both the exponential and Weibull distributions seem to fit well the waiting time and patience time distributions, respectively. Thus, we chose parameters based on the fit for the urgent level, which is the middle severity level.

Specifically, the two simulation settings consist of samples from exponential and Weibull distributions in which the waiting time has a smaller mean then the patience time mean, as was observed in the case study. In the first setting, following the data from the case study, a sample was taken from the model in which the patience time T follows an exponential distribution with expectation of 16 h, and the waiting time W follows an exponential distribution with expectation of 2 h. In the second setting a sample was taken from a model in which the patience time T follows a Weibull distribution with scale 16 and shape 1.5, which closely related to the observed data; and where the waiting time W follows an exponential distribution with expectation of 2 h as before. In both settings, the unknown probability of announcement is q(t)=exp(−t). Taking the probability of announcement to be the increasing function q(t)=1−exp(−t) or the constant function q(t)=0.5 yields similar results which are omitted. Moreover, we experimented with additional numerical values. The behavior and conclusions, as reported here, remain consistent across these experiments.

**FIGURE 2:**
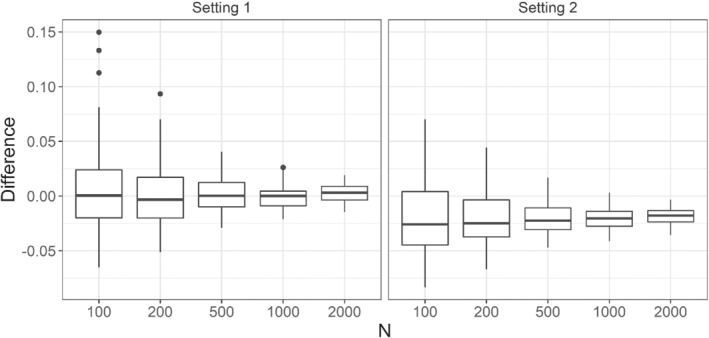
The difference between the semiparametric estimator of $\theta_{0}$ and $\theta_{0}$. Setting 1: The patience time T follows an exponential distribution with expectation of 16 h and the waiting time W follows and exponential distribution with expectation of 2 h. Setting 2: The patience time T follows a Weibull distribution with scale 16 and shape 1.5 while the waiting time W follows an exponential distribution with expectation of 2 h

In each setting, we calculated the semiparametric estimator for the scale of T for five different sample sizes (N=100,200,500,1000,2000). For each sample size, we repeated the simulation 100 times. When using the semiparametric method, it was assumed that both T and W follow an exponential distribution with unknown parameters. Note that this assumption holds for the first setting but does not hold for the second one. In other words, the second setting is carried out under a misspecified model. The results are shown in Figure 2.

**FIGURE 3:**
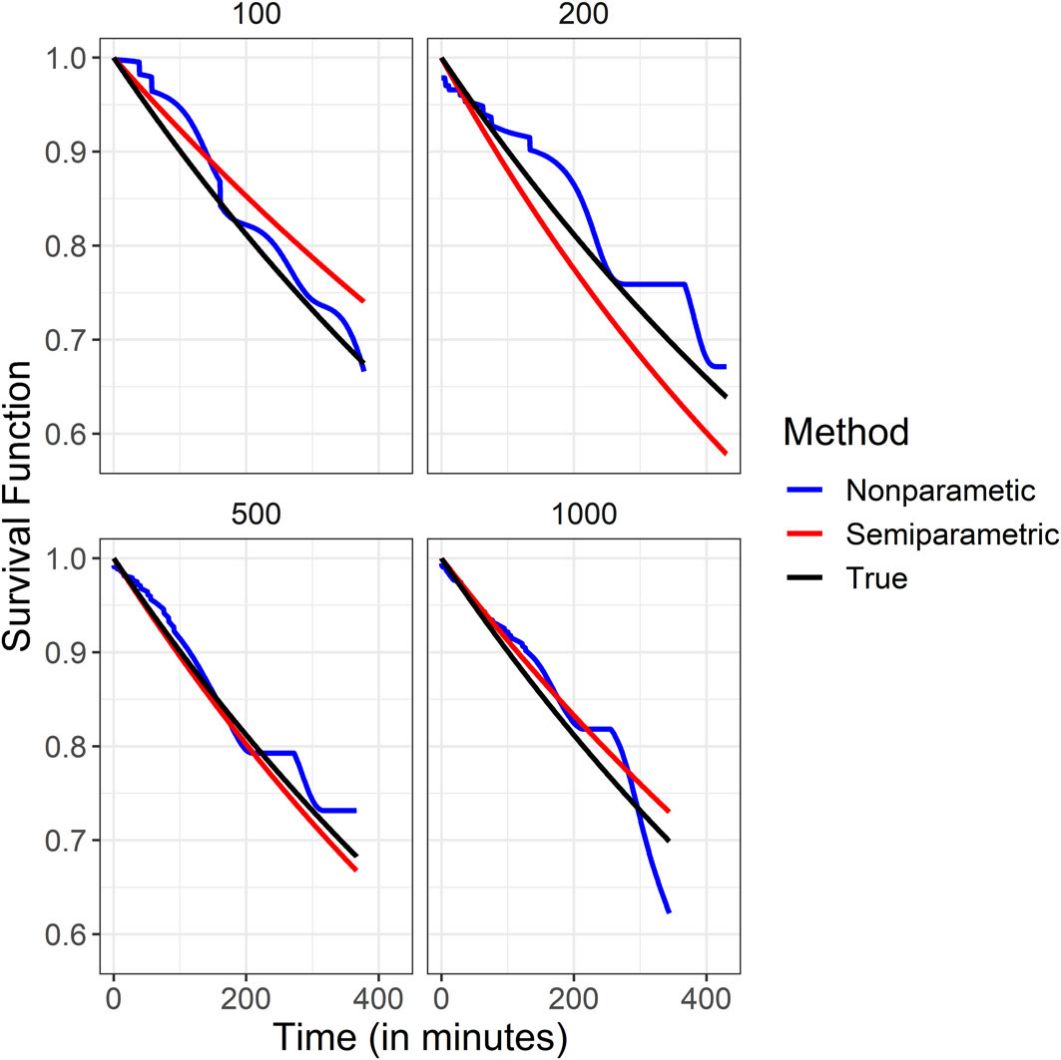
Setting 1. The blue, red, and black curves represent the nonparametric, semiparametric, and true survival functions, respectively, for N=100,200,500, and 1000

**FIGURE 4:**
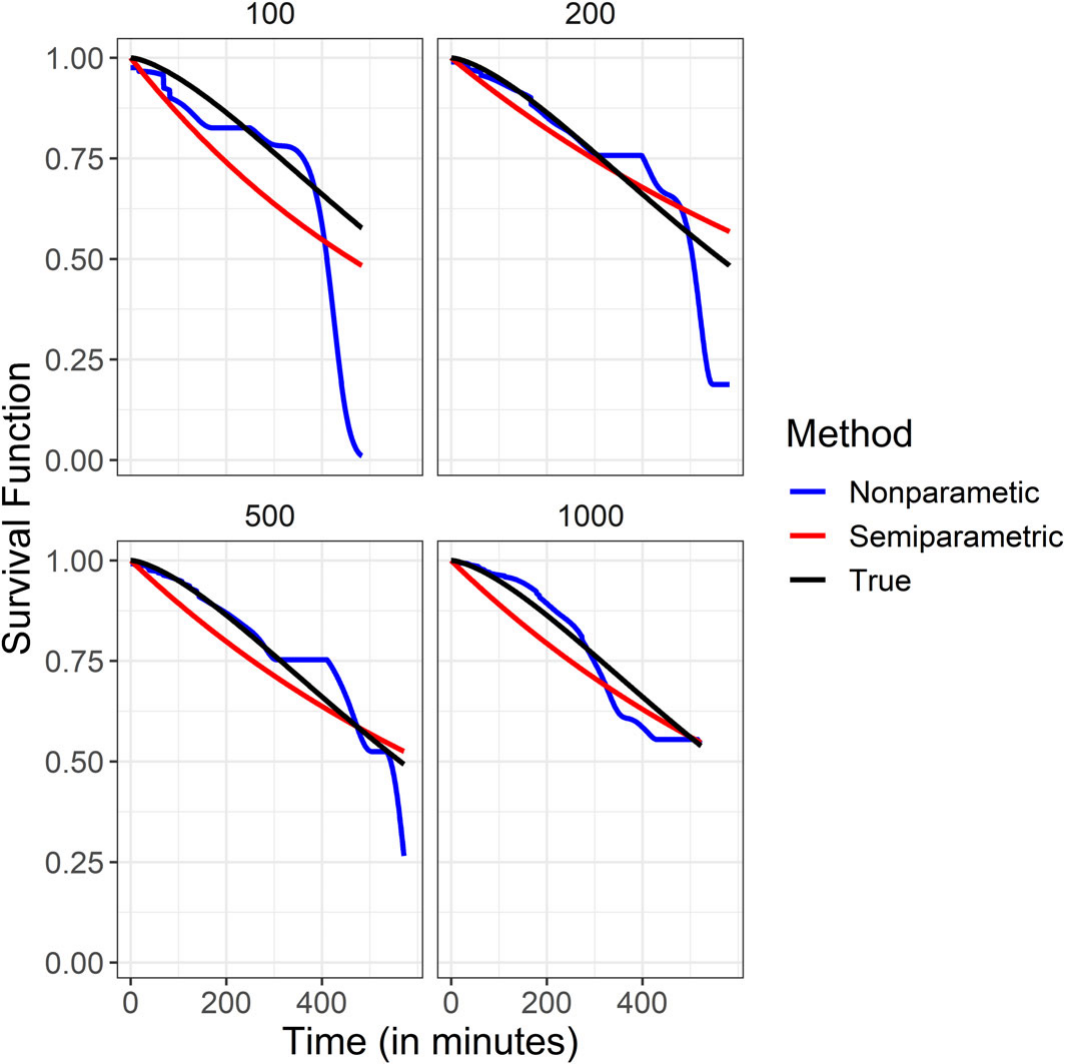
Second setting. The blue, red, and black curves represent the nonparametric, semiparametric, and true survival functions, respectively

We compare ${\hat{\bar{F}}}_{n}$, the estimator of the survival function of T, to the true survival function ${\bar{F}}_{0}$. For the semiparametric estimation, ${\hat{\bar{F}}}_{n}(t)=\exp(-\hat{\theta }t)$, while for the nonparametric estimator ${\hat{\bar{F}}}_{n}(t)$ is given by (A.4). The comparison is done using mean square error (MSE), which is defined by

$$MSE({\hat{\bar{F}}}_{n},{\bar{F}}_{0})\equiv \int_{-\infty }^{\infty }{\left\{{\hat{\bar{F}}}_{n}(t)-{\bar{F}}_{0}(t)\right\}}^{2}f_{0}(t)dt\;,$$

where $f_{0}$ is the density of T. The semiparametric and nonparametric survival function estimators are demonstrated in Figures 3 and 4. Figure 3 represents the results of the first setting in which T follows an exponential distribution with scale 13 and W follows an exponential distribution with scale 2. Figure 4 represents the results of the second setting in which T follows a Weibull distribution with scale 13 and shape 1.5, and W follows an exponential distribution with scale 2. Summaries of the MSE are given in Table 1. Not surprisingly, for Setting 1, since the semiparametric model is correct, the MSE is smaller for the semiparametric estimator. Similarly, since in Setting 2 the semiparametric model is incorrect, the MSE is smaller for the nonparametric estimator.

**TABLE 1:** MSE for Settings 1 and 2

	Setting 1: Exponential							Setting 2: Weibull					
	Semiparametric			Nonparametric				Semiparametric			Nonparametric		
N	Mean	Median	Std	Mean	Median	Std		Mean	Median	Std	Mean	Median	Std
100	1.014	0.432	1.44	1.428	0.804	1.812		0.474	0.228	0.618	0.216	0.114	0.264
200	0.51	0.21	0.828	1.062	0.672	1.11		0.414	0.252	0.492	0.072	0.036	0.12
500	0.162	0.084	0.198	0.462	0.288	0.474		0.378	0.294	0.288	0.03	0.018	0.03
1000	0.078	0.042	0.102	0.246	0.186	0.186		0.342	0.312	0.204	0.018	0.012	0.018
2000	0.054	0.03	0.066	0.168	0.132	0.12		0.348	0.318	0.132	0.012	0.0006	0.012

*Note*: The table summarizes the MSE that was calculated (100 times) for each of the sample sizes. For Setting 1, the patience time T follows an exponential distribution with expectation of 16 h and the waiting time W follows an exponential distribution with expectation of 2 h. In Setting 2 the patience time T follows a Weibull distribution with scale 16 and shape 1.5, while the waiting time W follows an exponential distribution with expectation of 2 h. The estimates are given in minutes. As can be seen the nonparametric estimator responded with a lower MSE.

## 7 CASE STUDY

As leaving without being seen by a physician may have a strong effect on patient well‐being and satisfaction, estimating the time that patients are willing to wait in the ED is an important and challenging question.^19^, ^25^ While there has been considerable research in this field,^6^, ^7^, ^8^, ^9^ due to the special structure of the data, the duration that a potential patient is willing to wait for ED service has not been thoroughly investigated.

**FIGURE 5:**
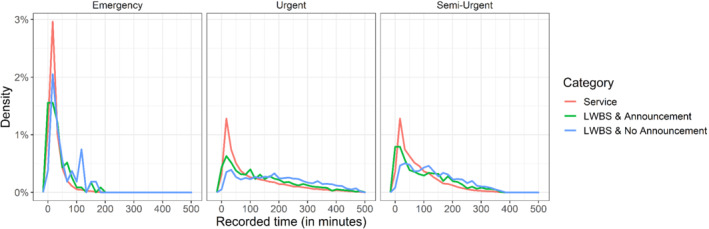
The distribution of the observed time stratified by acuity levels and category

We analyze data from all patient presentations to triage at an urban, academic, adult‐only ED with visits in calendar year 2008. This data was used for the analysis in Wiler et al.^19^ The data consist of the waiting time of patients arriving at the emergency room stratified by acuity levels. We focused on the three main levels of acuity: emergency, urgent, and semi‐urgent. For each acuity level, we categorized each visit into one of the three categories: received service, left without being seen and announced, and left without being seen and did not announce. We considered only patients that were not served upon arrival, or left without waiting at all. The characteristics of the dataset appear in Table 2. As can be seen, they are considerably fewer emergency visits and only 1.2% of these left without being seen. In comparison, in the urgent and semi‐urgent acuity levels, about 10% left without being seen. The distribution of the observed times for each acuity levels, stratified by the patient's category, appears in Figure 5. Overall, the distribution of the three categories is similar in each acuity level.

**TABLE 2:** The characteristics of the different visits stratified by acuity level

	Emergency	Urgent	Semi‐urgent
n	8579	36 249	26 036
Category (%)			
Service	8478 (98.8%)	32 607 (90.0%)	23 788 (91.4%)
LWBS & announcement	69 (0.8%)	1908 (5.3%)	1019 (3.9%)
LWBS & no announcement	32 (0.4%)	1734 (4.8%)	1229 (4.7)%
Mean observed time (SD)	25.11 (24.26)	111.73 (108.21)	82.18 (72.92)

**FIGURE 6:**
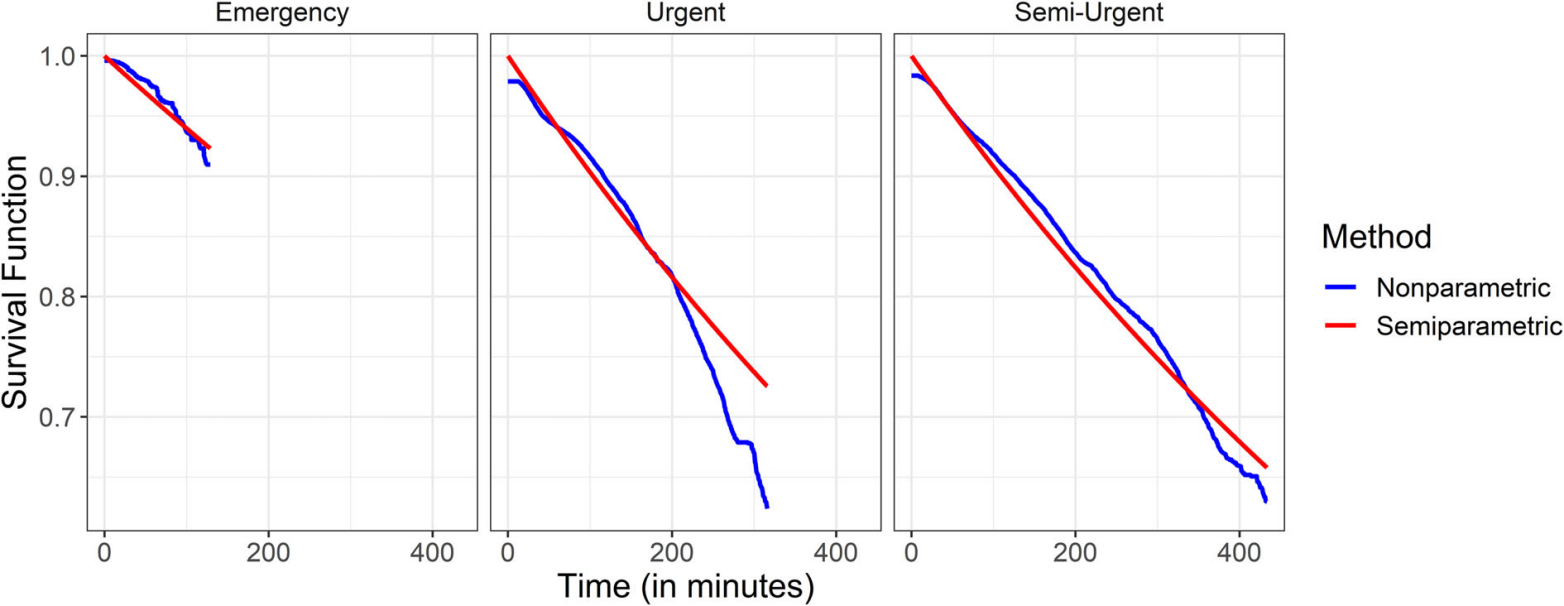
Compression of the nonparametric and semiparametric estimators for the survival of the patience time by different levels of acuity

**FIGURE 7:**
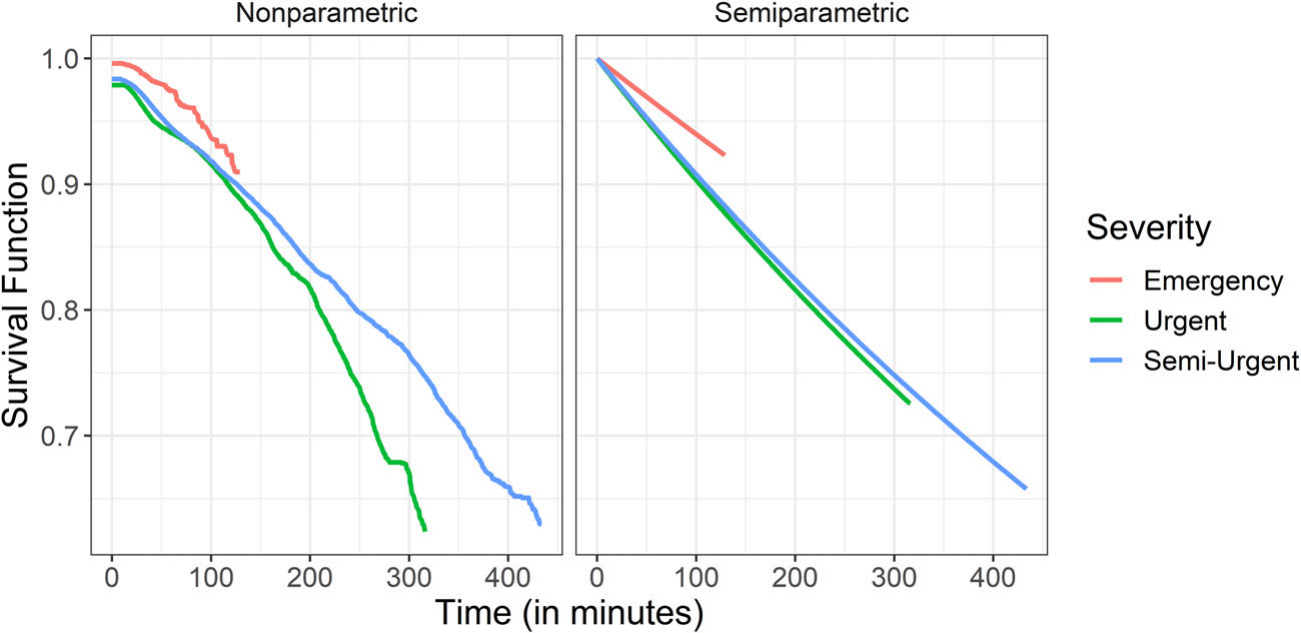
Compression of the estimator for the survival function of the patience time at the three different levels of severity

We analyzed the data using the semiparametric and nonparametric estimators for the distribution of the patience time proposed in Sections 4 and 5. Since our model assumes that all patients follow the same distribution, we calculated the estimators for each level of acuity separately. The data consist of the triple variables $\left(U_{i},\Delta_{i},Y_{i}\right)$ described in Section 3 such that each observation is categorized to one of the three possible categories. The results of these estimators are given in Figures 6 and 7. As can be seen from Figure 6, the results of the semiparametric and nonparametric estimators agree, which suggests that modeling the patience time using the exponential distribution is reasonable. Figure 7 shows that the patience times are stochastically ordered by levels of acuity. In other words, patients at the severe acuity level are less probable to lose patience than patients at the urgent level, who in turn are less prone to lose patience than patients at the semi‐urgent level, as expected.

## 8 DISCUSSION

In this article, we consider survival data that combine observed, right‐censored, and left‐censored data. The setting we analyzed was that of patients who wait for treatment in an ED, where some patients may leave without being seen. We proposed both semiparametric and nonparametric estimators for the distribution of the patience time.

Using simulation, we showed that when the semiparametric model holds, the semiparametric estimator estimates the patience time well. However, when the model is misspecified, the nonparametric estimator behaves better. While in our case study, both estimators behave similarly, it is of importance to further investigate when each of these estimators is preferable. So far, no baseline covariates were given. Novel semiparametric and nonparametric estimators are needed for addressing settings that include baseline covariates.

## CONFLICT OF INTEREST

The authors declare no potential conflict of interests.

## Data Availability

Due to privacy and confidentiality issues, research data are not shared.

## Proofs

### A.1 Proof of Lemma 1

$$\begin{array}{ll} pr(U\leq t,𝒞=1) & =pr(U\leq t,W\leq T)\\ & =pr(W\leq t,W\leq T)\\ & =\int_{0}^{t}pr(W\leq T|W=s)g(s)ds\\ & =\int_{0}^{t}pr(s\leq T)g(s)ds\\ & =\int_{0}^{t}g(s)\bar{F}(s)ds,\; \end{array}$$

where in the fourth equality we use the independence between W and (Y,T).

This establishes (i). For (ii), we have

$$\begin{array}{ll} pr(U\leq t,𝒞=2) & =pr(U\leq t,Y=1,T<W)\\ & =pr(T\leq t,Y=1,T<W)\\ & =\int_{0}^{t}pr(s<W|Y=1,T=s)q(s)f(s)ds\\ & =\int_{0}^{t}pr(s<W)q(s)f(s)ds\\ & =\int_{0}^{t}q(s)f(s)\bar{G}(s)ds, \end{array}$$

where in the fourth equality we use the independence between W and (Y,T).

Finally, for (iii),

$$\begin{array}{ll} P(U\leq t,𝒞=3) & =pr(U\leq t,Y=0,T<W)\\ & =pr(W\leq t,Y=0,T<W)\\ & =\int_{0}^{t}pr(Y=0,T<s|W=s)g(s)ds\\ & =\int_{0}^{t}g(s)pr(Y=0,T<s)ds\\ & =\int_{0}^{t}g(s)\int_{0}^{s}pr(Y=0\;|\;T=x)f(x)dxds\\ & =\int_{0}^{t}g(s)\int_{0}^{s}\left\{1-q(x)\right\}f(x)dxds, \end{array}$$

where in the fourth equality we use the independence between W and (Y,T).

### A.2 Proof of Theorem 1

The log of the full likelihood is

$$\begin{array}{ll} ł(D;\theta ,\gamma ) & =\overset{n}{\underset{i=1}{\sum }}[\left(1-\Delta_{i}\right)\left(\log g(U_{i};\gamma )+\log\bar{F}(U_{i};\theta )\right)\\ & \;+\Delta_{i}Y_{i}\left(\log q(U_{i})+\log f(U_{i};\theta )+\log\bar{G}(U_{i};\gamma )\right)\\ & \;+\;\Delta_{i}(1-Y_{i})\left\{\log g(U_{i};\gamma )+\log\int_{0}^{U_{i}}(1-q(s))f(s;\theta )ds\right\}]. \end{array}$$

Given the data D,

(A1)
$$\frac{1}{n}l(D;\theta ,\gamma )={\mathbb{P}}_{n}\left\{m_{\gamma }(U,\Delta ,Y)+c(U,\Delta ,Y;\theta )\right\}\equiv \frac{1}{n}\overset{n}{\underset{i=1}{\sum }}\left\{m_{\gamma }(U_{i},\Delta_{i},Y_{i})+c(U_{i},\Delta_{i},Y_{i};\theta )\right\},$$

where $m_{\gamma }:{\mathbb{R}}^{+}\times {\left\{0,1\right\}}^{2}\to \mathbb{R}$ is defined by

$$m_{\gamma }(u,\delta ,y)\equiv (1-\delta )\log g(u;\gamma )+\Delta y\log\bar{G}(u;\gamma )+\delta (1-y)\log g(u;\gamma ),$$

and

$$\begin{array}{ll} c(u,\delta ,y;\theta ) & \equiv (1-\delta )\log\bar{F}(u;\theta )\\ & \;+\delta y\left(\log q(u)+\log f(u;\theta )\right)+\delta (1-y)\log\int_{0}^{u}\left\{1-q(s)\right\}f(s;\theta )ds. \end{array}$$

From Assumption A1 we obtain that, for each θ∈Θ, $\arg\max_{\gamma \in \Gamma }l(D;\gamma ,\theta )=\arg\max_{\gamma \in \Gamma }{\mathbb{P}}_{n}(m_{\gamma })$. The γ that maximizes L(D;θ,γ) does not depend on the value of θ or the function p. Define $M_{n}(\gamma )\equiv {\mathbb{P}}_{n}m_{\gamma }$ and $M(\gamma )\equiv Pm_{\gamma }$. If, for a general function h, Ph≡∫h(x)dP(x) and ${\mathbb{P}}_{n}h\equiv n^{-1}\sum_{i=1}^{n}h(X_{i}$) then by Assumptions A1–A3, theorem 5.7 in van der Vaart^26^ can be applied. Therefore ${\hat{\gamma }}_{n}\to \gamma_{0}$, in probability, which concludes the proof of (i).

Given the data D, the term $\frac{\partial l(D;\theta ,\gamma )}{\partial \gamma }$ is a function of γ and does not depend on the unknown function p. We also have

$$\frac{1}{n}\frac{\partial l(D;\theta ,\gamma )}{\partial \gamma }={\mathbb{P}}_{n}\psi_{\gamma }(U,\Delta ,Y)\equiv \frac{1}{n}\overset{n}{\underset{i=1}{\sum }}\psi_{\gamma }(U_{i},\Delta_{i},Y_{i}),$$

where $\psi_{\gamma }:{\mathbb{R}}^{+}\times {\left\{0,1\right\}}^{2}\to \mathbb{R}$ is defined as

$$\psi_{\gamma }(u,\delta ,y)\equiv (1-\delta )\frac{\frac{\partial }{\partial \gamma }g(u;\gamma )}{g(u;\gamma )}-\delta y\frac{\frac{\partial }{\partial \gamma }\bar{G}(u;\gamma )}{\bar{G}(u;\gamma )}+\delta (1-y)\frac{\frac{\partial }{\partial \gamma }g(u;\gamma )}{g(u;\gamma )}.$$

By Assumptions A1–A3, $\psi_{\gamma }$ satisfies the conditions of theorem 5.41 in van der Vaart^26^ and, therefore,

$$\sqrt{n}({\hat{\gamma }}_{n}-\gamma_{0})=-{\left(P{\dot{\psi }}_{\gamma_{0}}\right)}^{-1}\frac{1}{\sqrt{n}}\overset{n}{\underset{i=1}{\sum }}\psi_{\gamma_{0}}(U_{i},\Delta_{i},Y_{i})+o_{p}(1),$$

where ${\dot{\psi }}_{\gamma }(x)=\frac{\partial }{\partial \gamma }\psi_{\gamma }(x)$. Hence ${\hat{\gamma }}_{n}$ is a linear asymptotically normal (LAN) estimator with influence function $\varphi \equiv -{\left(P{\dot{\psi }}_{\gamma_{0}}\right)}^{-1}\psi_{\gamma_{0}}$ . From all of the above we get that (ii) is proved with $V_{\gamma_{0}}={\left(P{\dot{\psi }}_{\gamma_{0}}\right)}^{-1}P\psi_{\gamma_{0}}\psi_{\gamma_{0}}^{t}{\left(P{\dot{\psi }}_{\gamma_{0}}\right)}^{-1}$.

To prove (iii), note that due to the term $\log\int_{0}^{U_{i}}(1-q(s))f(s;\theta )ds$ that appears in l(D;θ,γ), the term $\frac{\partial l(D;\theta ,\gamma )}{\partial \theta }$ depends on the unknown function p. We therefore consider a partial likelihood function such that its derivative with respect to θ does not depend on p. The partial likelihood that satisfies this request is the partial likelihood of 𝒞=1:

$$\overset{n}{\underset{i=1}{\prod }}\left\{\frac{g(U_{i};\gamma )\bar{F}(U_{i};\theta )}{\int_{0}^{\infty }g(s;\gamma )\bar{F}(s;\theta )ds}\right\}{}^{1-\Delta_{i}}.$$

The log of the partial likelihood is

$${ł}_{partial}(D;\theta ,\gamma )=\overset{n}{\underset{i=1}{\sum }}\left(1-\Delta_{i}\right)\left\{\log g(U_{i};\gamma )+\log\bar{F}(U_{i};\theta )-\log\int_{0}^{\infty }g(s;\gamma )\bar{F}(s;\theta )ds\right\}.$$

Given the data D, the term $l_{partial}(D;\theta ,\gamma )$ is a function only of the parameters θ and γ. We also have

$$\frac{1}{n}l_{partial}(D;\theta ,\gamma )={\mathbb{P}}_{n}r_{\theta ,\gamma }(U,\Delta ,Y)\equiv \frac{1}{n}\overset{n}{\underset{i=1}{\sum }}r_{\theta ,\gamma }(U_{i},\Delta_{i},Y_{i}),$$

where $r_{\theta ,\gamma }:{\mathbb{R}}^{+}\times {\left\{0,1\right\}}^{2}\to \mathbb{R}$ is given by

$$r_{\theta ,\gamma }(U,\Delta ,Y)\equiv \left(1-\Delta \right)\left\{\log g(U;\gamma )+\log\bar{F}(U;\theta )-\log\int_{0}^{\infty }g(s;\gamma )\bar{F}(s;\theta )ds\right\}.$$

Define $M_{n}(\theta ,\gamma )\equiv {\mathbb{P}}_{n}r_{\theta ,\gamma }$, and $M(\theta ,\gamma )\equiv Pr_{\theta ,\gamma }$. Then, theorem 5.7 in van der Vaart^26^ can be applied. Therefore $\left({\hat{\theta }}_{n},{\hat{\gamma }}_{n}\right)\to \left(\theta_{0},\gamma_{0}\right)$ in probability, and in particular ${\hat{\theta }}_{n}\to \theta_{0}$ in probability, and (iii) is proven.

In order to prove (iv), note that

$$\frac{1}{n}\frac{\partial l_{partial}(D;\theta ,\gamma )}{\partial \gamma }={\mathbb{P}}_{n}\phi_{\theta ,\gamma }(U,\Delta ,Y)\equiv \frac{1}{n}\overset{n}{\underset{i=1}{\sum }}\phi_{\theta ,\gamma }(U_{i},\Delta_{i},Y_{i}),$$

where $\phi_{\theta ,\gamma }:{\mathbb{R}}^{+}\times {\left\{0,1\right\}}^{2}\to \mathbb{R}$ is defined as

$$\phi_{\theta ,\gamma }(u,\delta ,y)\equiv (1-\delta )\left\{\frac{\frac{\partial }{\partial \theta }\bar{F}(u;\theta )}{\bar{F}(u;\theta )}-\frac{\frac{\partial }{\partial \theta }\int_{0}^{\infty }g(s;\gamma )\bar{F}(s;\theta )ds}{\int_{0}^{\infty }g(s;\gamma )\bar{F}(s;\theta )ds}\right\}.$$

Using Assumptions A1–A3, together from theorem 5.41 in van der Vaart,^26^ we obtain that

$$\sqrt{n}({\hat{\gamma }}_{n}-\gamma_{0})=\frac{1}{\sqrt{n}}\overset{n}{\underset{i=1}{\sum }}\left\{-{\left(P{\dot{\psi }}_{\gamma_{0}}\right)}^{-1}\psi_{\gamma_{0}}(U_{i},\Delta_{i},Y_{i})\right\}+o_{p}(1).$$

Define $\Phi_{n}(\theta ,\gamma )\equiv {\mathbb{P}}_{n}\phi_{\theta ,\gamma }$ and note that $\Phi (\theta_{0},\gamma_{0})\equiv P\phi_{\theta_{0},\gamma_{0}}=0$ (since under the true parameters $P\phi_{\theta_{0},\gamma_{0}}=\frac{\partial }{\partial \theta }\int dh_{0}=\frac{\partial }{\partial \theta }1=0$, where $dh_{0}$ is the true distribution of category 1).

By Taylor's theorem,

$$\begin{array}{ll} & 0=\Phi_{n}({\hat{\theta }}_{n},{\hat{\gamma }}_{n})=\Phi_{n}(\theta_{0},\gamma_{0})+{\left\{\frac{\partial }{\partial \theta }\Phi_{n}(\theta_{0},\gamma_{0})\right\}}^{T}({\hat{\theta }}_{n}-\theta_{0})+{\left\{\frac{\partial }{\partial \phi }\Phi_{n}(\theta_{0},\gamma_{0})\right\}}^{T}({\hat{\gamma }}_{n}-\gamma_{0})+o_{p}\left(n^{-1/2}\right)\\ \Rightarrow & 0=\sqrt{n}\Phi_{n}(\theta_{0},\gamma_{0})+{\left\{\frac{\partial }{\partial \theta }\Phi_{n}(\theta_{0},\gamma_{0})\right\}}^{T}\sqrt{n}\left({\hat{\theta }}_{n}-\theta_{0}\right)+{\left\{\frac{\partial }{\partial \gamma }\Phi_{n}(\theta_{0},\gamma_{0})\right\}}^{T}\sqrt{n}\left\{{\hat{\gamma }}_{n}-\gamma_{0}\right\}+o_{p}(1).\\ & =\sqrt{n}\Phi_{n}(\theta_{0},\gamma_{0})+{\left\{\frac{\partial }{\partial \theta }\Phi_{n}(\theta_{0},\gamma_{0})\right\}}^{T}\sqrt{n}\left({\hat{\theta }}_{n}-\theta_{0}\right)\\ & \;-{\left\{\frac{\partial }{\partial \gamma }\Phi_{n}(\theta_{0},\gamma_{0})\right\}}^{T}{\left(P{\dot{\psi }}_{\gamma_{0}}\right)}^{-1}\frac{1}{\sqrt{n}}\overset{n}{\underset{i=1}{\sum }}\psi_{\gamma_{0}}(U_{i},\Delta_{i},Y_{i})+o_{p}(1)\\ & =\frac{1}{\sqrt{n}}\overset{n}{\underset{i=1}{\sum }}\left[\phi_{\theta_{0},\gamma_{0}}(U_{i},\Delta_{i},Y_{i})-{\left\{\frac{\partial }{\partial \gamma }\Phi_{n}(\theta_{0},\gamma_{0})\right\}}^{T}{\left(P{\dot{\psi }}_{\gamma_{0}}\right)}^{-1}\psi_{\gamma_{0}}(U_{i},\Delta_{i},Y_{i})\right]\\ & \;+{\left\{\frac{\partial }{\partial \theta }\Phi_{n}(\theta_{0},\gamma_{0})\right\}}^{T}\sqrt{n}({\hat{\theta }}_{n}-\theta_{0})+o_{p}(1). \end{array}$$

Elementary arithmetic leads to

$$\sqrt{n}\left({\hat{\theta }}_{n}-\theta_{0}\right)=-{\left(EE^{T}\right)}^{-1}\frac{1}{\sqrt{n}}\overset{n}{\underset{i=1}{\sum }}\left\{\phi_{\theta_{0},\gamma_{0}}(U_{i},\Delta_{i},Y_{i})-B^{T}{\left(P{\dot{\psi }}_{\gamma_{0}}\right)}^{-1}\psi_{\gamma_{0}}(U_{i},\Delta_{i},Y_{i})\right\}+o_{p}(1),$$

where $B\equiv \frac{\partial }{\partial \gamma }\Phi (\theta_{0},\gamma_{0})$, and $E\equiv \frac{\partial }{\partial \theta }\Phi (\theta_{0},\gamma_{0})$. Hence, ${\hat{\theta }}_{n}$ is a LAN estimator with the influence function

$$\varphi =-{\left(EE^{T}\right)}^{-1}\frac{1}{\sqrt{n}}\left\{\phi_{\theta_{0},\gamma_{0}}-B^{T}{\left(P{\dot{\psi }}_{\gamma_{0}}\right)}^{-1}\psi_{\gamma_{0}}\right\}.$$

Summarizing, $\sqrt{n}({\hat{\theta }}_{n}-\theta_{0})\to N(0,S_{\theta_{0},\gamma_{0}})$ in distribution, where $S_{\theta_{0},\gamma_{0}}=P\varphi \varphi^{T}$, hence, (iv) is proven.

### A.3 Proof of Lemma 2

We use similar arguments to those in the proof of the convergence of the Nelson‐Aalen estimator to a cumulative hazard function, see Kosorok,^23^ page 240. Hence we have that

$$\sqrt{n}\left\{\begin{array}{c} {\hat{N}}_{n}(t)-N(t)\\ {\hat{Y}}_{n}(t)-Y(t) \end{array}\right\}=O_{p}(1),$$

where N(t)=pr(Y=1,T≤W,T≤t)andY(t)=pr(U≥t). Since, by Section 3,

{U>t}={Y=0,W>t}∪{Y=1,W>t,T>t}.

Hence,

$$\begin{array}{ll} pr(U>t) & =pr(Y=0,W>t)+pr(Y=1,W>t,T>t)\\ & =pr(W>t)\left\{pr(Y=0)+pr(Y=1,T>t)\right\}\\ & =pr(W>t)(pr(Y=0)+pr(Y=1)-pr(Y=1,T\leq t))\\ & =pr(W>t)\left\{1-pr(Y=1,T\leq t)\right\}\\ & =\bar{G}(t)\left\{1-\int_{0}^{t}q(s)f(s)ds\right\}, \end{array}$$

where in the second equality we use the independence between W and (Y,T). By Lemma 1

$$pr(Y=1,T\leq W,T\leq t)=\int_{0}^{t}q(s)f(s)\bar{G}(s)ds.$$

Using the continuity of the derivative operator and of the integral operator, we get that

$${\hat{D}}_{n}(t)\to \int_{0}^{t}\frac{q(s)f(s)\bar{G}(s)}{\bar{G}(s)\left\{1-\int_{0}^{s}q(x)f(x)ds\right\}}ds,$$

in probability. Note that

$$\begin{array}{ll} & \int_{0}^{t}\frac{\bar{G}(s)q(s)f(s)}{\bar{G}(s)\left\{1-\int_{0}^{s}q(x)f(x)dx\right\}}ds=\int_{0}^{t}\frac{q(s)f(s)}{\left\{1-\int_{0}^{s}q(x)f(x)dx\right\}}ds\\ & \;=-\int_{0}^{t}\frac{\partial }{\partial s}\log\left\{1-\int_{0}^{s}q(x)f(x)dx\right\}ds=-\log\left\{1-\int_{0}^{t}q(s)f(s)ds\right\}. \end{array}$$

Hence, by the delta method, see Kosorok^23^ chapter 12.2.2.2, we get that

$${\hat{D}}_{n}(t)\to -\log\left\{1-\int_{0}^{t}q(s)f(s)ds\right\},$$

in probability, with convergence at rate $n^{1/2}$. Since y=−log(1−x)⇔x=1−exp(−y) and by the continuous mapping theorem, see theorem 7.7 of Kosorok,^23^ we get that $\hat{A}(t)=1-\exp(-{\hat{D}}_{n}(t))$ is an estimator of $\int_{0}^{t}q(s)f(s)ds$, at the rate of $n^{1/2}$ as desired.

### A.4 Proof of Theorem 2

For the proof of Theorem 2, we need the following lemma, which is elementary hence stated without proof.

Lemma 3
*Let* ${\left(a_{n}\right)}_{n=1}^{\infty },{\left(b_{n}\right)}_{n=1}^{\infty }$ *be positive sequences. If* $X_{n}-X=O_{p}(a_{n})$ *and*

$Y_{n}-Y=O_{p}(b_{n})$ *, as well as* P(|X|>l)=1 *for some* l>0 *. Then we have*:

1. $X_{n}+Y_{n}-(X+Y)=O_{p}(a_{n}\vee b_{n}),$
2. $X_{n}Y_{n}-XY=O_{p}(a_{n}\vee b_{n}),$
3. $\frac{1}{X_{n}}-\frac{1}{X}=O_{p}(a_{n}).$

Proof of Theorem 2 Recall that

$${\hat{F}}_{n}(t)=\frac{\hat{pr}(Y=0,W<T){\hat{r}}_{3}(t)+\hat{pr}(T\leq W){\hat{r}}_{1}(t)\hat{A}(t)}{\hat{pr}(Y=0,W<T){\hat{r}}_{3}(t)+\hat{pr}(T\leq W){\hat{r}}_{1}(t)},$$

is an estimator of F(t). For all t>0,

$$\hat{pr}(Y=0,W<T)-pr(Y=0,W<T)=O_{p}(n^{-1/2})\;,$$

and

$$\hat{pr}(T\leq W)-pr(T\leq W)=O_{p}(n^{-1/2})\;,$$

as both are empirical distribution estimators. By chapter 1.7 of Tsybakov,^24^ for all t>0, ${\hat{r}}_{j}(t)-r_{j}(t)=O_{p}(n^{-\beta /(2\beta +1)})$, for j=1,3. By Lemma 2, $\hat{A}(t)-A(t)=O_{p}(n^{-1/2})$. By Lemma 3.ii

$$\hat{pr}(Y=0,W<T){\hat{r}}_{3}(t)-pr(Y=0,W<T){\hat{r}}_{3}(t)=O_{p}(n^{-\beta /(2\beta +1)})\;,$$

and

$$\hat{pr}(T\leq W){\hat{r}}_{1}(t)\hat{A}(t)-pr(T\leq W){\hat{r}}_{1}(t)A(t)=O_{p}(n^{-\beta /(2\beta +1)})\;.$$

Therefore, by Lemma 3.i,

$$\begin{array}{ll} & \hat{pr}(Y=0,W<T){\hat{r}}_{3}(t)+\hat{pr}(T\leq W){\hat{r}}_{1}(t)\hat{A}(t)-pr(Y=0,W<T)r_{3}(t)-pr(T\leq W)r_{1}(t)A(t)\\ & \;=O_{p}(n^{-\beta /(2\beta +1)})\;. \end{array}$$

Similarly,

$$\begin{array}{ll} & \hat{pr}(Y=0,W<T){\hat{r}}_{3}(t)+\hat{pr}(T\leq W){\hat{r}}_{1}(t)-pr(Y=0,W<T)r_{3}(t)-pr(T\leq W)r_{1}(t)\\ & \;=O_{p}(n^{-\beta /(2\beta +1)}). \end{array}$$

By Lemma 3.iii,

$$\begin{array}{ll} & \frac{1}{\hat{pr}(Y=0,W<T){\hat{r}}_{3}(t)+\hat{pr}(T\leq W){\hat{r}}_{1}(t)}-\frac{1}{pr(Y=0,W<T)r_{3}(t)+pr(T\leq W)r_{1}(t)}\\ & \;=O_{p}(n^{-\beta /(2\beta +1)}). \end{array}$$

By Lemma 3.i again,

$$\begin{array}{ll} & \frac{\hat{pr}(Y=0,W<T){\hat{r}}_{3}(t)+\hat{pr}(T\leq W){\hat{r}}_{1}(t)\hat{A}(t)}{\hat{pr}(Y=0,W<T){\hat{r}}_{3}(t)+\hat{pr}(T\leq W){\hat{r}}_{1}(t)}-\frac{pr(Y=0,W<T)r_{3}(t)+pr(T\leq W)r_{1}(t)A(t)}{pr(Y=0,W<T)r_{3}(t)+pr(T\leq W)r_{1}(t)}\\ & \;=O_{p}(n^{-\beta /(2\beta +1)}). \end{array}$$

In other words, ${\hat{F}}_{n}(t)-F(t)=O_{p}(n^{-\beta /(2\beta +1)})$, which complete the proof of Theorem 2.

### A.5 Details on Example 1

Assuming that T follows an exponential distribution with rate θ and W follows an exponential distribution with rate gamma then the likelihood L(D;θ,γ) is

$$\begin{array}{ll} \overset{n}{\underset{i=1}{\prod }} & (\left\{\gamma \exp(-\gamma U_{i})\exp(-\theta U_{i})\right\}{}^{1-\Delta_{i}}\left\{q(U_{i})\theta \exp(-\theta U_{i})\exp(-\gamma U_{i})\right\}{}^{\Delta_{i}Y_{i}}\\ & \times {\left[\gamma \exp(-\gamma U_{i})\int_{0}^{U_{i}}\left\{1-q(s)\right\}\theta \exp(-\theta s)\right]}^{\Delta_{i}(1-Y_{i})}). \end{array}$$

Hence,

$$\begin{array}{ll} & \log L(D;\theta ,\gamma )\\ & \;=\overset{n}{\underset{i=1}{\sum }}(1-\Delta_{i})(\log\gamma -\gamma U_{i})+\overset{n}{\underset{i=1}{\sum }}\Delta_{i}Y_{i}(-\gamma U_{i})+\overset{n}{\underset{i=1}{\sum }}\Delta_{i}(1-Y_{i})(\log\gamma -\gamma U_{i})+C(U_{1},U_{2},...,U_{n};\theta )\\ & \;=(n-\overset{n}{\underset{i=1}{\sum }}\Delta_{i}Y_{i})\log\gamma -\gamma \overset{n}{\underset{i=1}{\sum }}U_{i}+C(U_{1},U_{2},...,U_{n};\theta ). \end{array}$$

Therefore,

$$\frac{\partial \log L(D;\theta ,\gamma )}{\partial \gamma }=\frac{n-\sum_{i=1}^{n}\Delta_{i}Y_{i}}{\gamma }-\overset{n}{\underset{i=1}{\sum }}U_{i},$$

and hence, the value of γ which maximize L(D;θ,γ) is ${\hat{\gamma }}_{n}=\frac{n-\sum_{i=1}^{n}\Delta_{i}Y_{i}}{\sum_{i=1}^{n}U_{i}}$.

The partial likelihood $L_{partial}(D;\theta ;\gamma )$ of category 𝒞=1,

$$\begin{array}{ll} & \overset{n}{\underset{i=1}{\prod }}{\left\{\frac{g(U_{i};\gamma )\bar{F}(U_{i};\theta )}{\int_{0}^{\infty }g(s;\gamma )\bar{F}(s;\theta )ds}\right\}}^{1-\Delta_{i}}\\ & \;=\overset{n}{\underset{i=1}{\prod }}{\left\{\frac{\gamma \exp(-\gamma U_{i})\exp(-\theta U_{i})}{\int_{0}^{\infty }\gamma \exp(-\gamma s)\exp(-\theta s)ds}\right\}}^{1-\Delta_{i}}=\overset{n}{\underset{i=1}{\prod }}{\left\{\frac{\gamma \exp\left(-U_{i}(\gamma +\theta )\right)}{\int_{0}^{\infty }\gamma \exp\left(-s(\gamma +\theta )\right)ds}\right\}}^{1-\Delta_{i}}\\ & \;=\overset{n}{\underset{i=1}{\prod }}{\left\{\frac{\gamma \exp\left(-U_{i}(\gamma +\theta )\right)}{\frac{\gamma }{\gamma +\theta }}\right\}}^{1-\Delta_{i}}=\overset{n}{\underset{i=1}{\prod }}{\left[(\gamma +\theta )\exp\left(-U_{i}(\gamma +\theta )\right)\right]}^{1-\Delta_{i}}. \end{array}$$

Hence,

$$\log L_{partial}(D;\theta ;\gamma )=\overset{n}{\underset{i=1}{\sum }}(1-\Delta_{i})\left(\log(\gamma +\theta )-U_{i}(\gamma +\theta )\right).$$

Therefore,

$$\frac{\partial \log L_{partial}(D;\theta ,\gamma )}{\partial \theta }=\overset{n}{\underset{i=1}{\sum }}(1-\Delta_{i})(\frac{1}{\gamma +\theta }-U_{i}).$$

The semiparametric estimator for θ is the maximizer of $L_{partial}(D;\theta ,{\hat{\gamma }}_{n})$ by θ which is

$${\hat{\theta }}_{n}=\frac{\sum_{i=1}^{n}(1-\Delta_{i})}{\sum_{i=1}^{n}U_{i}(1-\Delta_{i})}-{\hat{\gamma }}_{n}=\frac{\sum_{i=1}^{n}(1-\Delta_{i})}{\sum_{i=1}^{n}U_{i}(1-\Delta_{i})}-\frac{n-\sum_{i=1}^{n}\Delta_{i}Y_{i}}{\sum_{i=1}^{n}U_{i}}.$$
